# Paeonolide as a Novel Regulator of Core-Binding Factor Subunit Alpha-1 in Bone-Forming Cells

**DOI:** 10.3390/ijms22094924

**Published:** 2021-05-06

**Authors:** Kyung-Ran Park, Joon Yeop Lee, Myounglae Cho, Jin Tae Hong, Hyung-Mun Yun

**Affiliations:** 1 Department of Oral and Maxillofacial Pathology, School of Dentistry, Kyung Hee University, Seoul 02447, Korea; rudfks282@naver.com; 2 National Institute for Korean Medicine Development, Gyeongsan 38540, Korea; chool9090@nikom.or.kr (J.Y.L.); meanglae@nikom.or.kr (M.C.); 3 College of Pharmacy and Medical Research Center, Chungbuk National University, Chungbuk 28160, Korea; jinthong@chungbuk.ac.kr

**Keywords:** *Paeonia suffruticosa*, paeonolide, osteoblast differentiation, bone mineralization, ERK1/2, RUNX2, Cbfa1

## Abstract

*Paeonia suffruticosa* has been extensively used as a traditional medicine with various beneficial effects; paeonolide (PALI) was isolated from its dried roots. This study aimed to investigate the novel effects and mechanisms of PALI in pre-osteoblasts. Here, cell viability was evaluated using an MTT assay. Early and late osteoblast differentiation was examined by analyzing the activity of alkaline phosphatase (ALP) and by staining it with Alizarin red S (ARS). Cell migration was assessed using wound healing and Boyden chamber assays. Western blot and immunofluorescence analyses were used to examine the intracellular signaling pathways and differentiation proteins. PALI (0.1, 1, 10, 30, and 100 μM) showed no cytotoxic or proliferative effects in pre-osteoblasts. In the absence of cytotoxicity, PALI (1, 10, and 30 μM) promoted wound healing and transmigration during osteoblast differentiation. ALP staining demonstrated that PALI (1, 10, and 30 μM) promoted early osteoblast differentiation in a dose-dependent manner, and ARS staining showed an enhanced mineralized nodule formation, a key indicator of late osteoblast differentiation. Additionally, low concentrations of PALI (1 and 10 μM) increased the bone morphogenetic protein (BMP)–Smad1/5/8 and Wnt–β-catenin pathways in osteoblast differentiation. Particularly, PALI (1 and 10 μM) increased the phosphorylation of ERK1/2 compared with BMP2 treatment, an FDA-approved drug for bone diseases. Furthermore, PALI-mediated early and late osteoblast differentiation was abolished in the presence of the ERK1/2 inhibitor U0126. PALI-induced RUNX2 (Cbfa1) expression and nuclear localization were also attenuated by blocking the ERK1/2 pathway during osteoblast differentiation. We suggest that PALI has biologically novel activities, such as enhanced osteoblast differentiation and bone mineralization mainly through the intracellular ERK1/2-RUNX2 signaling pathway, suggesting that PALI might have therapeutic action and aid the treatment and prevention of bone diseases, such as osteoporosis and periodontitis.

## 1. Introduction

Abnormalities in bone formation are characterized in bone diseases such as osteoporosis and periodontitis [1]. The number of patients suffering from these diseases increases with their age [2]. Osteoporosis affects the elderly over the age of 65 years and causes increased bone fragility, fracture risk, and a reduction in bone mass [3,4]. Periodontitis occurs in adults, young people, and children, and leads to periodontal tissue destruction and alveolar bone loss [5,6]. 

Osteoblasts play important roles in controlling skeletal integrity by forming new bone and remodeling old bone, and the dysfunction of osteoblasts in proliferation, migration, and differentiation contributes to the pathogenesis of bone diseases [7,8,9,10]. Osteoblasts originate from mesenchymal stem cells (MSCs), multipotent stem cells found in the bone marrow, that are important for maintaining homeostasis in tissues, such as cartilage, bone, and fat [11,12]. MSCs differentiate into osteoblasts by osteogenic factors, such as bone morphogenetic proteins (BMPs) and Wnts [11,12]. Osteoblasts lead to the synthesis of bone-specific proteins and bone matrix mineralization, and some osteoblasts are surrounded by the mineralized bone matrix and remain osteocytes [12,13,14,15,16]. 

A variety of compounds, such as paeonol, paeonoside, paeonolide, paeoniflorin, and mudanpioside H, have been identified in *Paeonia suffruticosa* [17,18]. Many studies have reported that *P. suffruticosa* has therapeutic effects on inflammatory, neurological, cancer, and cardiovascular diseases [19]. Paeonol, the main compound isolated from *P. suffruticosa,* possesses anti-inflammatory and antioxidant activities, which inhibit osteoclastogenesis and alveolar bone loss through the Nrf2/NF–κB/NFATc1 signaling pathway in ligation-induced periodontitis [20]. Paeonol also inhibits the RANKL-induced ERK, p38, and NF-κB activation in macrophages to suppress osteoclastogenesis and ovariectomy-induced osteoporosis [21]. However, the biological effects of PALI and its potential effects on osteoblasts remain unclear.

In the present study, we isolated PALI from the dried roots of *P. suffruticosa*, investigated the biological effects of PALI in osteoblasts, and demonstrated the mechanism of action of PALI in osteoblast differentiation and bone matrix mineralization.

## 2. Results

### 2.1. PALI Enhances Cell Migration without Cytotoxicity in Pre-Osteoblasts 

**Figure 1:**
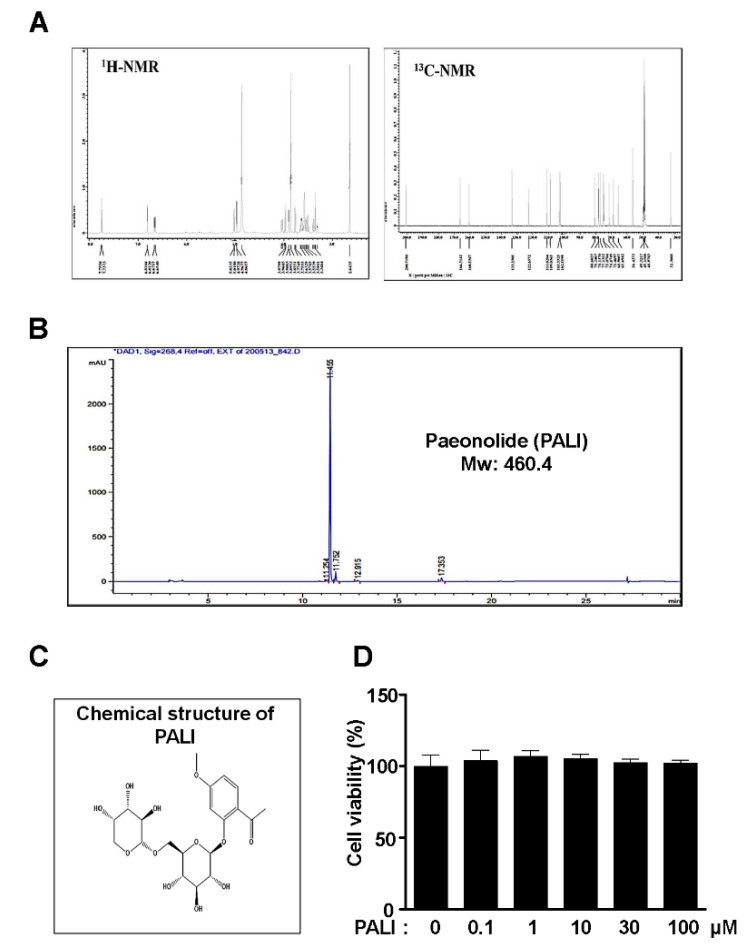
Effects of paeonolide (PALI) isolated from the roots of *P. suffruticosa* on cytotoxicity and proliferation in pre-osteoblasts. (**A**) ^1^H and ^13^C NMR spectra of PALI from the roots of *P. suffruticosa*. (**B**) HPLC chromatogram of PALI. (**C**) Chemical structure of PALI. (**D**) Pre-osteoblasts were treated with PALI at concentrations ranging from 0.1 to 100 μM for 24 h, and cell viability (%) was measured using an MTT assay. Data are representative of the results of three independent experiments and values are expressed as mean ± SEM.

PALI was isolated from the dried roots of *P. suffruticosa* and analyzed using the NMR spectra and an HPLC chromatogram (Figure 1A,B). PALI is a glycoside containing a non-reducing end α-l-arabinopyranoside (Figure 1C). To examine the biological effects of PALI on cell toxicity and proliferation against pre-osteoblasts, the cells were treated with PALI (0.1, 1, 10, 30, and 100 μM) for 24 h and cell viability (%) was measured using an MTT assay. At concentrations ranging from 0.1 to 100 μM, PALI did not affect cytotoxicity and proliferation against pre-osteoblasts (Figure 1D).

**Figure 2:**
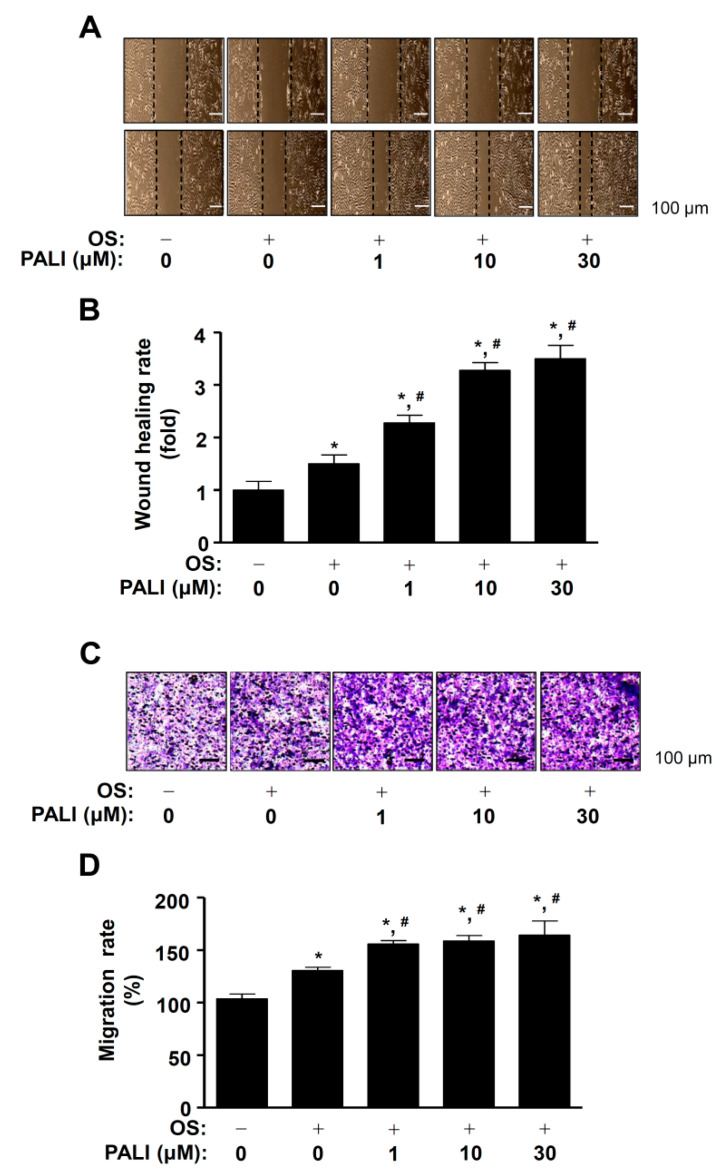
Effects of paeonolide (PALI) on cell migration during osteoblast differentiation. (**A**,**B**) Pre-osteoblasts were treated with PALI at concentrations ranging from 1 to 30 μM during osteogenic supplement medium (OS)-induced osteoblast differentiation for 24 h. The wound healing assay was performed and results examined under a phase contrast microscope (**A**), and wound healing rate (fold) was measured and is expressed as a bar graph normalized to that of the control (**B**). (**C**,**D**) The Boyden chamber assay was carried out and observed under a phase contrast microscope (**C**). The bar graph of migration rate (%) was normalized to that of the control (**D**). Data are representative of the results of three independent experiments and values are expressed as mean ± SEM. *, *p* < 0.05 indicates a statistically significant difference compared with the control; #, statistically significant difference compared with OS (*p* < 0.05).

Cell migration increased during early osteoblast differentiation and adhesiveness increases at a later stage [22]. Next, we examined whether PALI affected cell migration during osteoblast differentiation. Osteoblast differentiation was induced using an OS medium containing 50 μg/mL l-ascorbic acid and 10 mM β-glycerophosphate in the presence or absence of 1 to 30 μM PALI for 24 h, after which cell migration was investigated using a wound healing assay. The area of the wound measured immediately after scratching was significantly recovered by treatment with PALI in a dose-dependent manner (Figure 2A,B). To clarify PALI-mediated cell migration, the effect of PALI on cell migration was evaluated using a Boyden chamber assay that revealed that PALI significantly increased transmigration past the Nucleopore filter in a dose-dependent manner (Figure 2C,D).

### 2.2. PALI Promotes Osteoblast Differentiation and Mineralized Nodule Formation

**Figure 3:**
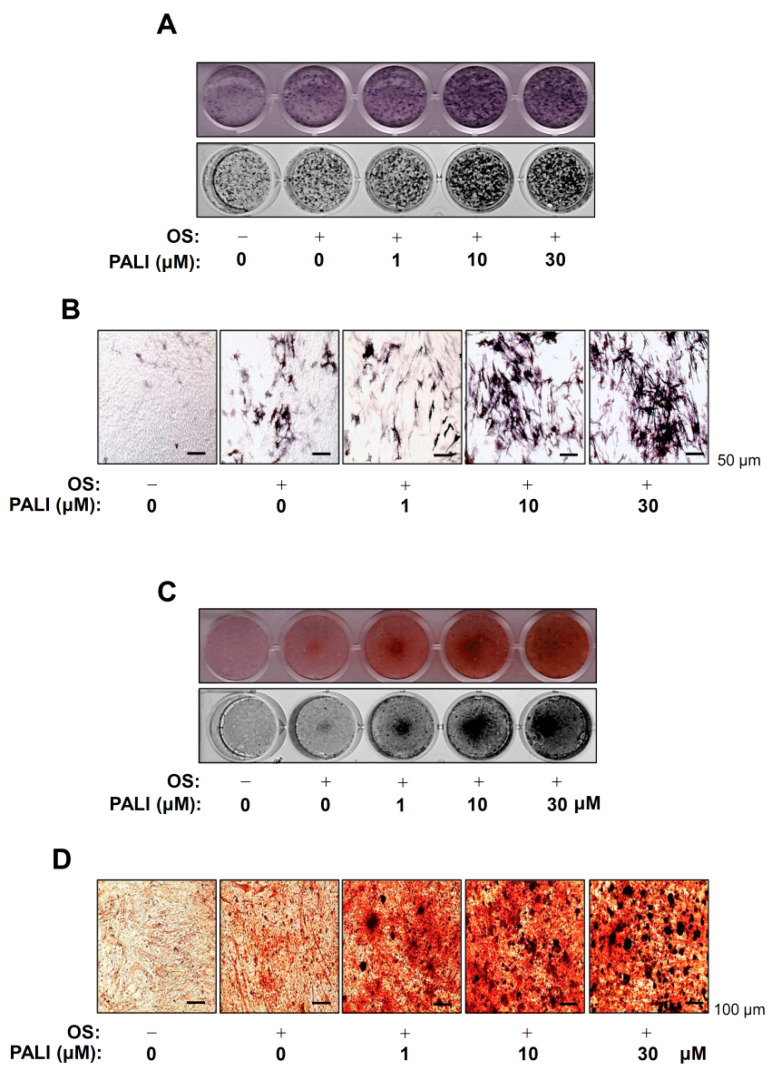
Effects of paeonolide (PALI) on the osteoblast differentiation of pre-osteoblasts. (**A**,**B**) Pre-osteoblasts were treated with the indicated concentrations of PALI during osteogenic supplement medium (OS)-induced osteoblast differentiation for seven days. Alkaline phosphatase (ALP) staining was analyzed under a scanner (*upper*) and a colorimetric detector (*bottom*) (**A**), and ALP-positive cells were visualized using a light microscope (**B**). Scale bar: 50 μm. (**C**,**D**) The mineralized nodule formation was examined using Alizarin red S (ARS) staining at 14 days. The ARS staining was analyzed using a scanner (*upper*) and a colorimetric detector (*bottom*) (**C**), and the mineralization nodules were visualized using a light microscope (**D**). Scale bar: 100 μm. Data are representative of the results of three independent experiments and values are expressed as mean ± SEM.

To investigate the biological activities of PALI on the early osteoblast differentiation of pre-osteoblasts, we stimulated osteoblast differentiation using OS in the presence or absence of PALI (1–30 μM) for seven days and measured alkaline phosphatase (ALP) staining. The stains were observed using a digital camera and colorimetric detector. Treatment with PALI resulted in an increase in early osteoblast differentiation of pre-osteoblasts (Figure 3A). Consistent with these results, ALP-positive cells in response to PALI were also validated by observation under a light microscope (Figure 3B).

Next, we measured the degree of matrix mineralization using Alizarin red S (ARS) staining to assess late osteoblast differentiation. After inducing osteoblast differentiation in the presence or absence of 1 to 30 μM PALI for 14 days, we detected matrix mineralization using a scanner and colorimetric detector. As shown in Figure 3C, PALI increased the formation of mineralized nodules in a dose-dependent manner. PALI-mediated mineralized nodule formation was also confirmed by light microscopy (Figure 3D). Furthermore, PALI potentiated the expression of osteoblast-specific genes, including those encoding *Alp*, osteopontin (*Opn*), and osteocalcin (*Ocn*) (Supplementary Figure S1A–C).

### 2.3. PALI Increases the BMP2, Wnt3a, and MAPKs Pathways during Osteoblast Differentiation

**Figure 4:**
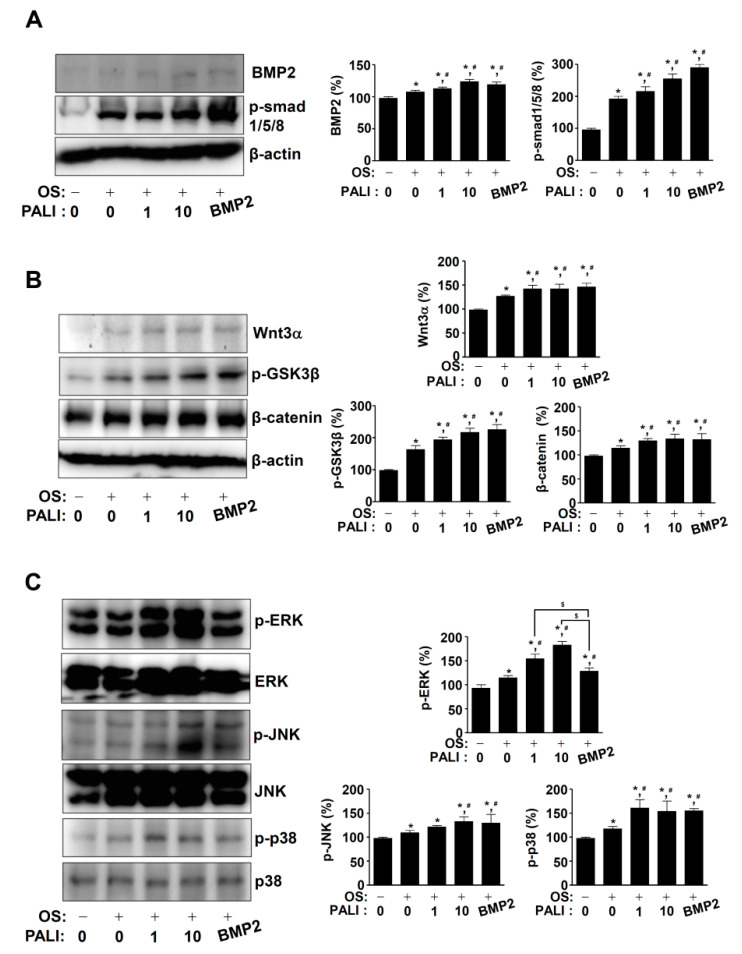
Effects of paeonolide (PALI) on BMP2, Wnt3, and MAPKs signaling during osteoblast differentiation. (**A**–**C**) After pre-osteoblasts were treated with PALI or BMP2 (100 ng/mL) during osteogenic supplement medium (OS)-induced osteoblast differentiation for 24 h, equal amounts of lysates were analyzed using Western blot analysis and detected with antibodies against BMP2, phospho-Smad1/5/8 (p-Smad1/5/8) (**A**); Wnt3a, phospho-GSK3β (p-GSK3β), and β-catenin (**B**); and phospho-ERK (p-ERK), ERK, phospho-JNK (p-JNK), JNK, phospho-p38 (p-p38), and p38 (**C**). β-actin was used as a loading control. BMP2 was used as a positive control. Data are representative of the results of three independent experiments. Bar graphs are represented as relative percentages of the control. Data are representative of the results of three independent experiments and values are expressed as mean ± SEM. *, *p* < 0.05 indicates a statistically significant difference compared with the control; #, statistically significant difference compared with OS (*p* < 0.05); $, statistically significant difference compared with the OS + PALI (*p* < 0.05).

To examine the mechanism of action of PALI in osteoblast differentiation, we examined the BMP2, Wnt3a, and MAPK signaling pathways using Western blotting. First, PALI-mediated osteoblast differentiation at concentrations of 1 and 10 μM increased BMP2 protein expression and the phosphorylation of Smad1/5/8 compared with that with OS (Figure 4A). Second, PALI treatment enhanced Wnt3a protein expression, phosphorylation of GSK3β, and protein level of β-catenin during osteoblast differentiation (Figure 4B). Third, we investigated the effects of PALI on MAPKs in osteoblast differentiation. At concentrations of 1 and 10 μM of PALI, the phosphorylation of ERK1/2, JNK, and p38 increased in a dose-dependent manner (Figure 4C). The main finding in the BMP2, Wnt3a, and MAPK pathways is that PALI obviously promotes the phosphorylation of ERK1/2 compared with that with BMP2 treatment (Figure 4).

### 2.4. PALI-Induced ERK1/2 Activation Enhances Osteoblast Differentiation by Regulating RUNX2 Expression during Osteoblast Differentiation

**Figure 5:**
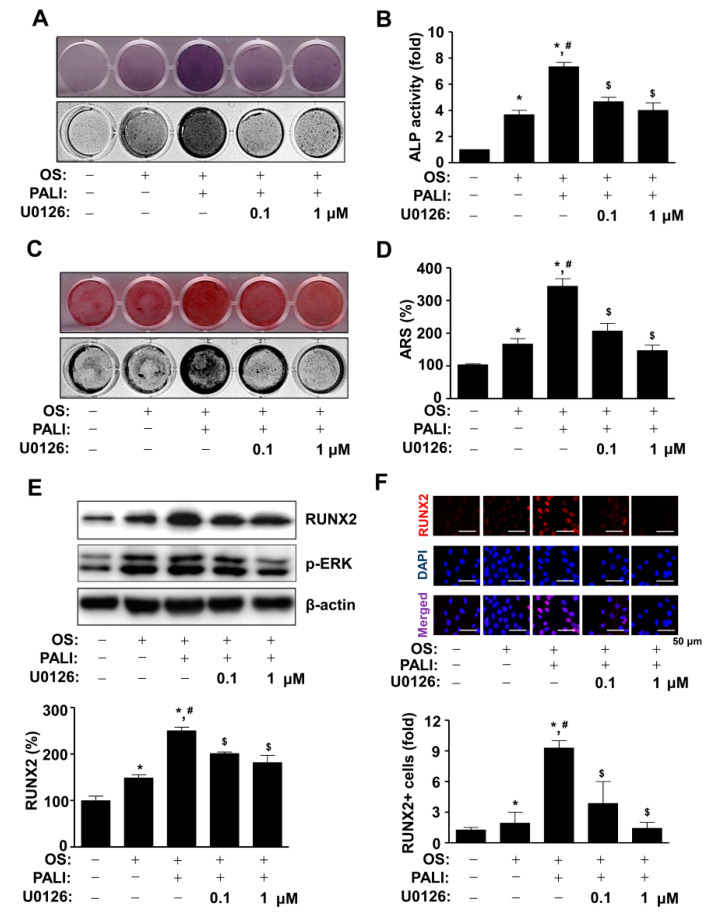
Effects of paeonolide (PALI)-induced ERK1/2 signals on osteoblast differentiation. (**A**,**B**) After pre-osteoblasts were treated with PALI (10 μM) in the presence or absence of U0126 (1 and 10 μM) during osteogenic supplement medium (OS)-induced osteoblast differentiation, alkaline phosphatase (ALP) staining was analyzed using a scanner (*upper*) and a colorimetric detector (*bottom*) (**A**), and the enzymatic activity of (ALP) was measured at 405 nm using a spectrophotometer and is expressed as a bar graph. (**C**,**D**) Alizarin red S (ARS) staining was analyzed using a scanner (upper) and a colorimetric detector (bottom) (**C**), and the intensity of mineralized nodule formation was quantified and is expressed as a bar graph (**D**). (**E**,**F**) Equal amounts of lysates were analyzed using Western blot analysis and detected with antibodies against RUNX2 and phospho-ERK (p-ERK). β-Actin was used as a loading control (**E**). The fixed cells were analyzed using immunofluorescence analysis and immunostained with antibody against RUNX2 (red). DAPI (blue) was used as a nuclear marker. The bottom panels show the merged images (purple) of the first and second panels. RUNX2+ cells (fold) were expressed as a bar graph. Scale bar: 50 μm. Data are representative of the results of three independent experiments and values are expressed as mean ± SEM. *, *p* < 0.05 indicates statistically significant difference compared with the control; #, statistically significant difference compared with OS (*p* < 0.05); $, statistically significant difference compared with the OS + PALI (*p* < 0.05).

To further investigate the role of PALI-induced ERK1/2 activation in osteoblast differentiation, pre-osteoblasts were treated with PALI in the presence or absence of an ERK1/2 inhibitor, U0126 (0.1 and 1 μM). Pretreatment with U0126 significantly inhibited PALI-mediated ALP staining and ALP enzymatic activity during osteoblast differentiation (Figure 5A,B). Subsequently, the formation of mineralized nodules in response to the treatment with PALI was attenuated by U0126 treatment (Figure 3D). The effect of PALI-induced ERK1/2 activation on osteoblast differentiation was also demonstrated by ARS staining and quantification (Figure 5C,D). As ERK1/2 controls RUNX2 (Cbfa1), which is a key transcription factor in osteoblast differentiation, we next examined whether PALI-induced ERK1/2 activation regulates RUNX2 expression during osteoblast differentiation. The concentration of U0126 (0.1 and 1 μM) abolished the phosphorylation of ERK1/2 during osteoblast differentiation (Figure 5E). Under these conditions, Western blot analysis indicated that pretreatment with U0126 significantly blocked PALI-stimulated RUNX2 expression (Figure 5E). Consistent with these findings, immunofluorescence analysis also demonstrated that U0126 attenuated the nuclear localization and expression of RUNX2 in response to PALI treatment during osteoblast differentiation (Figure 5F).

## 3. Discussion

Osteoblasts are the main target cells for the identification and development of anabolic drugs in bone diseases [23,24]. However, anabolic drugs are limited owing to their relatively high price and adverse effects [24,25,26,27]. Natural compounds from plants have received great attention in regard to treating bone diseases because they have been used to treat a variety of diseases and have shown fewer adverse effects [28,29,30]. Therefore, the identification of potential compounds is important for the effective and safe prevention and treatment of bone diseases. We previously reported evidence on the biological mechanisms and intracellular signaling of TMARg isolated from the roots of *Rubia cordifolia* Nakai. In the osteogenesis of pre-osteoblasts and mesenchymal precursors, we demonstrated that the alkaloid compound piperyline, from the fruits of *Piper nigrum* L., induces apoptosis and inhibits adhesion and migration in pre-osteoblasts through the apoptotic and Src/FAK signaling pathways, and subsequently suppresses osteoblast differentiation [31,32]. In the present study, we isolated PALI from the dried roots of *P. suffruticosa* and demonstrated its biological effects on osteoblast differentiation and matrix formation.

Osteoblast differentiation is required for the synthesis and secretion of bone proteins and mineralization of bone tissues [14,15,16]. In addition, the migration of osteoblasts from the bone marrow, periosteum, surrounding tissues, and circulating blood plays an important role in the appropriate bone formation and bone tissue repair [22,33,34]. The present results revealed that PALI promoted cell migration during osteoblast differentiation. In addition, PALI-mediated osteoblast differentiation increased ALP levels and mineralized nodule formation. ALP is upregulated and regulates specific osteoblast genes during early osteoblast differentiation, whereas the extracellular bone matrix is mineralized by calcium deposition during late osteoblast differentiation [11,35,36]. These data suggest that PALI has biological effects on osteoblast migration and differentiation, demonstrating its potential for bone formation and repair.

Osteogenic growth factors BMPs and Wnts mainly activate Smad1/5/8, β-catenin, and MAPKs to increase the protein levels and activities of RUNX2, which is essential for osteoblast commitment and differentiation [12,13,14,15,16,37,38]. Abnormal regulation of RUNX2 was reported to impair bone development and bone ossification by reducing of osteocalcin expression [39]. In the present study, we showed that PALI promoted the BMP2-Smad1/5/8, β-catenin, and MAPK pathways in osteoblast differentiation. Compared with BMP2 effects, PALI had a dominant effect on the activation of ERK1/2 among Smad1/5/8, β-catenin, p38, and JNK. We also found that PALI-induced RUNX2 expression and localization in the nucleus were also abolished by the ERK1/2 inhibitor U0126. Furthermore, our data demonstrated that the inhibition of ERK1/2 attenuated PALI-mediated osteoblast differentiation. It was reported that MAPKs, such as ERK1/2, p38, and JNK, control the stabilization and activity of RUNX2 by facilitating acetylation and preventing proteasomal degradation [40]. Inhibition of ERK1/2 reduced osteocalcin expression through the transcriptional activity of RUNX2 [41]. ERK1/2 is also required for the nuclear localization of RUNX2 [42]. Thus, the findings of our study suggest that PALI has anabolic effects on osteoblast differentiation and matrix mineralization by regulating ERK1/2-mediated RUNX2 expression.

In conclusion, we originally demonstrated the anabolic effects of PALI, including migration, differentiation, and mineralization, on osteoblasts based on molecular and cellular mechanisms. Anabolic drugs alleviate bone diseases via osteoblast differentiation and bone formation [23,24]. Therefore, our findings suggest that PALI might be a phytotherapeutic compound for the development of anabolic drugs for the treatment of bone diseases, such as osteoporosis and periodontal disease.

## 4. Materials and Methods

### 4.1. General Material for Extraction and Isolation from Paeonia suffruticosa

Dried roots of *Paeonia suffruticosa* were purchased from Human Herb Co., Ltd. (Gyoengsan, Korea). The methanol (MeOH), *n*-hexane (Hx), ethyl acetate (EtOAc), dichloromethane (CH_2_Cl_2_), and butyl alcohol (BuOH) were purchased from Duksan Chemical Co. (Anseong, Korea). For column chromatography, we used silica gel 60 (230–400 mesh, ASTM, Merck, Darmstadt, Germany) and ODS-A (12 nm, S-150 m, YMC, Tokyo, Japan). The NMR spectra were recorded on a JEOl ECA-500 spectrometer operating at 500 MHz for the ^1^H and 125 MHz for the ^13^C NMR spectrum. High-performance liquid chromatography (HPLC) spectra were recorded on an Agilent 1200 series (Agilent Technologies, Santa Clara, CA, USA) equipped with a photodiode array detector (PDA) and an evaporative light scattering detector (ELSD).

### 4.2. Extraction and Isolation

The dried roots of *Paeonia suffruticosa* (2 kg) were extracted with 95% MeOH for 2 h (3 × 500 mL). The MeOH extract (386.3 g) was then suspended in 1000 mL of distilled water and the solvent partitioned with *n*-hexane, EtOAc, and BuOH. The EtOAc-soluble fraction (43.8 g) was isolated using a silica gel column eluted with a stepwise gradient of CH_2_Cl_2_ and MeOH (99:1–50:1) to yield 13 fractions (PSE 1–13). The fraction PSE 2 was re-chromatographed on a silica gel column with an isocratic solvent condition of *n*-hexane and EtOAc (10:1) to obtain 11 fractions (PSE 2-1–PSE 2-11). The PSE 2-7 subfraction was separated by reversed-phase column chromatography (ODS-A) and eluted with 70% aqueous MeOH (*v*/*v*) to obtain paeonolide (580 mg). The chemical structure of paeonolide (purity: 92.1%) was determined on the basis of spectroscopic data and comparison with the previous literature, and identified as a PALI [43].

### 4.3. Paeonolide (PALI)

Light yellow powder; EI-MS *m*/*z* = 460.43 [M]^+^, molecular formula C_20_H_28_O_12_; ^1^H-NMR (500 MHz, CD_3_OD) δ: 7.75 (1H, d, *J* = 8.9 Hz, H-6), 6.81 (1H, d, *J* = 2.3 Hz, H-5), 6.66 (1H, d, *J* = 8.9 Hz, H-3), 5.03 (1H, d, *J* = 7.8 Hz, H-1′), 4.97 (1H, d, *J* = 2.6 Hz, H-1″), 2.64 (3H, s, H-8), 4.04 (1H, d, *J* = 9.8 Hz, H-6′b), 3.97 (1H, d, *J* = 9.7 Hz, H-6′a), 3.89 (1H, d, *J* = 2.6 Hz, H-2′), 3.86 (3H, s, OCH3), 3.77 (1H, d, *J* = 9.7 Hz, H-2″), 3.49-3.66 (6H, m, H-5′, 3″, 5″b, 4′, 3′, 5″a), 3.38 (1H, m, H-4′); ^13^C-NMR (125 MHz, CD_3_OD) δ: 200.7 (C-7), 166.3 (C-4), 160.5 (C-2), 133.2 (C-6), 122.7 (C-1), 111.0 (C-5), 109.0 (C-1″), 103.3 (C-3), 102.6 (C-1′), 80.6 (C-3′), 78.2 (C-5′), 78.1 (C-3″), 77.1 (C-2′), 75.1 (C-4′), 74.9 (C-2″), 71.5 (C-6′), 68.9 (C-4″), 65.7 (C-5″), 56.4 (OCH_3_), 32.3 (C-8).

### 4.4. Pre-Osteoblast Culture

MC3T3-E1 pre-osteoblasts (CRL-2593) purchased from the American Type Culture Collection (ATCC) (Manassas, VA, USA) were cultured in α-minimum essential medium (α-MEM) (WELGEME, Inc., Seoul, Republic of Korea) without L-ascorbic acid (Sigma-Aldrich, St. Louis, MO, USA) supplemented with 10% fetal bovine serum (FBS), penicillin (100 units/mL), and streptomycin (100 μg/mL) at 37 °C in a humidified atmosphere of 5% CO_2_ and 95% air as previously described [44].

### 4.5. Osteoblast Differentiation

Osteoblast differentiation was induced using osteogenic supplement medium (OS) containing 50 μg/mL L-ascorbic acid (L-AA) and 10 mM β-glycerophosphate (β-GP). The medium was replaced every two days during the incubation period as previously described [44].

### 4.6. MTT Assay

Cell viability and proliferation were assessed using a 3-(4,5-dimethylthiazol-2-yl)-2,5-diphenyl tetrazolium bromide (MTT) solution to detect NADH-dependent dehydrogenase activity, as previously described [45]. Formazan was dissolved in 100% DMSO, and absorbance was measured at a wavelength of 540 nm using the Multiskan GO Microplate Spectrophotometer (Thermo Fisher Scientific, Waltham, MA, USA).

### 4.7. Wound Healing and Boyden Chamber Assays

Cell migration was assessed using a wound healing assay as previously described [46]. Briefly, cells were wounded with a sterile 200 μL pipette tip, and the cells were incubated with PALI for 24 h at 37 °C in a humidified atmosphere of 5% CO_2_ and 95% air. Cell migration was also assessed with some modifications using the Boyden chamber assay as previously described [47]. Briefly, the Nucleopore filter was coated with Matrigel, and the cells were incubated in the Boyden chamber for 4 h, fixed in 10% formalin, and stained with 0.5% crystal violet. Cell images were visualized using a light microscope.

### 4.8. Alkaline Phosphatase (ALP) Staining Assay

Osteoblast differentiation was induced using OS containing 50 μg/mL L-AA and 10 mM β-GP with PALI (1, 10, and 30 μM) for seven days. ALP staining assay was performed as previously described [44]. Cells were fixed in 10% formalin, washed with distilled water, and incubated at 37 °C for 1 h in substrate solution for ALP reaction (Takara Bio Inc., Tokyo, Japan). The level of ALP staining was observed using a scanner and colorimetric detector (ProteinSimple Inc., Santa Clara, CA, USA).

### 4.9. ALP Activity Assay

Osteoblast differentiation was induced using OS containing 50 μg/mL L-AA and 10 mM β-GP with PALI (1, 10, and 30 μM) for seven days. ALP activity assay was performed as previously described [46].

### 4.10. Alizarin Red S (ARS) Staining

Osteoblast differentiation was induced using OS containing 50 μg/mL L-AA and 10 mM β-GP with PALI (1, 10, and 30 μM) for 14 days. ARS staining was performed as previously described [32]. Briefly, cells were fixed in 10% formalin, washed with distilled water, and stained with 2% Alizarin red S (pH 4.2) (Sigma-Aldrich, St. Louis, MO, USA) for 10 min. The level of ARS staining was observed using a scanner and a colorimetric detector (ProteinSimple Inc., Santa Clara, CA, USA).

### 4.11. Western Blot Analysis

Western blot analysis was performed as previously described [48]. Briefly, after sodium dodecyl sulfate-polyacrylamide gel electrophoresis, the proteins were transferred to polyvinylidene difluoride membranes (Millipore, Bedford, MA, USA). After blocking with 5% skimmed milk dissolved in Tris-buffered saline with 0.05% Tween 20 (TBST) for 1 h at room temperature, the immunoblots were incubated with the primary antibodies (1:1000) overnight at 4 °C, washed with 1× TBST, and then incubated with horseradish peroxidase (HRP)-conjugated secondary antibodies (1:5000, Jackson ImmunoResearch, West Grove, PA, USA) for 1 h at room temperature. Immunoreactive proteins were visualized using an enhanced chemiluminescence kit (Millipore, Bedford, MA, USA) in the ProteinSimple detection system (ProteinSimple Inc., Santa Clara, CA, USA).

### 4.12. Immunofluorescence

Immunofluorescence was performed as previously described [49]. Cells were fixed for 10 min at room temperature, permeabilized with 0.2% Triton X-100 in 1× PBS for 20 min, and blocked with 3% BSA diluted in 1× PBS for 1 h. The cells were incubated with anti-RUNX2 primary antibody (1:200, Cell Signaling Technology, Beverly, MA, USA) overnight at 4 °C. After washing three times in 1× PBS, the cells were incubated with Alexa Fluor 568-conjugated secondary antibody (1:500, Invitrogen, Carlsbad, CA, USA) for 2 h at room temperature, stained with DAPI (Sigma-Aldrich, St. Louis, MO, USA) for 10 min at room temperature, washed three times, and mounted on glass slides. Fluorescence images were captured using a confocal microscope (K1-Fluo Confocal Laser Scanning Microscope, Republic of Korea).

### 4.13. Statistical Analysis

Statistical analysis was performed using GraphPad Prism software version 5.0.0 for Windows (GraphPad Software Inc., San Diego, CA, USA; www.graphpad.com, accessed on 4 January 2021). Data are presented as mean ± standard error of the mean (SEM). Statistical differences between the groups were analyzed using a Student’s unpaired *t*-test. Statistical significance was set at *p* < 0.05.

## Supplementary Materials

The following are available online at https://www.mdpi.com/article/10.3390/ijms22094924/s1.

## Abbreviations

ALP	Alkaline phosphatase
ARS	Alizarin red S
β-GP	β-glycerophosphate
BMP	Bone morphogenetic protein
Cbfa1	core-binding factor subunit alpha-1
L-AA	L-ascorbic acid
MSCs	Mesenchymal stem cells
MTT	3-[4,5-dimethylthiazol-2-yl]-2,5-diphenyltetrazolium bromide
PALI	Paeonolide
OS	Osteogenic supplement medium
RUNX2	Runt-related transcription factor 2
