# Supplementary material for: Paeonolide as a Novel Regulator of Core-Binding Factor Subunit Alpha-1 in Bone-Forming Cells
Source: Int J Mol Sci. 2021 May 6;22(9):4924. doi: 10.3390/ijms22094924 (PMC8125120; doi:10.3390/ijms22094924)

Supplementary Figure legend

- **Supplementary Figure 1. Effects of PALI on osteoblast-specific genes. (A-C)** Cells were cultured in OS with PALI for 7 days. Total RNA was isolated, osteoblast-marker genes including *Alp* (A),*Opn* (B), *Ocn* (C) were analyzed, and then the target gene levels were normalized to β-actin. The data are representative of the results of three independent experiments and values are expressed as mean ± SEM. *, $p < 0.05$ indicates statistically significant difference, compared with the control. #: statistically significant difference compared with OS ($p < 0.05$).

Supplementary Figure 1

A

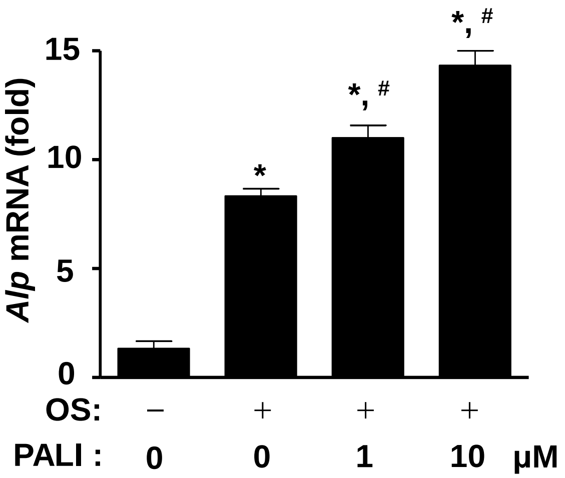

B

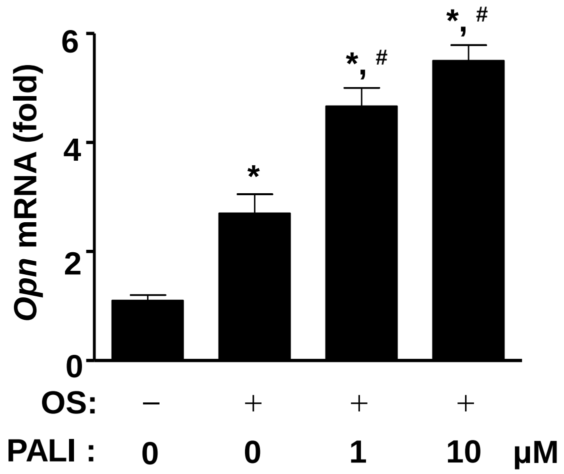

C

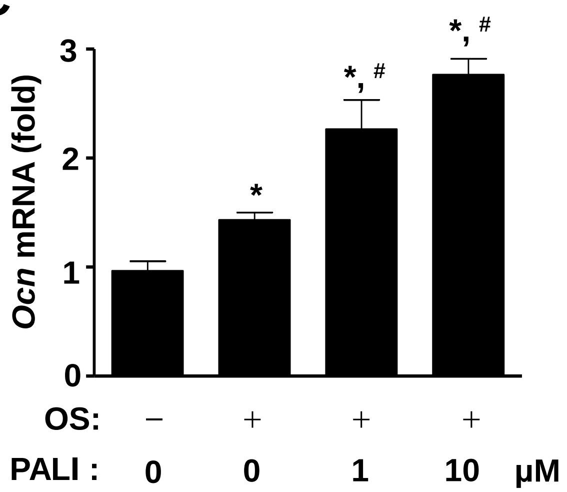

Supplement: Supplementary file 1 [file ijms-22-04924-s001.zip › ijms-1167181-supplementary.pdf]
